# Salines in Hernias

**Published:** 1917-04

**Authors:** G. Henri Bogart

**Affiliations:** Shelbyville, Ill.


### Salines in Hernias.

BY DR. G. HENRI BOGART, SHELBYVILLE, ILL.

That one should use, and use successfully, salines to control a hernia might not be considered as very ethical practice, particularly, when the exhibition of the remedy came through a layman, who had nothing, only common sense and reason for his guide.

En passant, wouldn't it be something fine, were we able to teach these two matters, common sense and reason, as curriculum features?

I was sitting in the writing room of a hotel when the landlord hurried to me saying, "Doctor, there's a gentleman up in No. —, who ought to have a doctor, but refuses; I wish that you would come up and see him."

Naturally, I declined, but on his continued urgency I finally went with him as a casual visitor.

The man was a traveler, who had arrived from a train, the middle of the afternoon, and who had immediately gone to bed.

He had an inguinal hernia, and on arrival he was white with the pain, while great drops of perspiration stood over his face.

When I went to his room I found him lying flat on his back, on a couch, drawn close to the hot water faucet.

He was applying hot compresses to the hernia, which was protuberant as an ordinary grape fruit.

He informed me that he had taken common epsom salts in half ounce doses, with abundance of water, every hour, and that he would soon have relief.

He said that for eight years he had cared for the rupture, without either operation, truss or binder, and that it had, in fact, proven a benefit to his general health. Previous to that time, he had been careless in his eating, but proceeding on the theory that any mechanical obstruction in the intestine would have to pass through the loop in the hernia, and so cause obstruction, he had chewed all his food to complete liquification. He would not eat an apple without paring it and cutting out the core; the bits of cartilage and the like in meats were trimmed from his food, and a regular habit of morning stools was cultivated.

At times, a large dose of Mag. Sulph. was taken with copious draughts of water in the morning, before breakfast, so as to flush the "pockets"—as he expressed it—of the colon.

I asked him how, with all this care, the present attack could have come about, and he told me that the previous evening he had eaten an orange in his room, and that without thinking he had eaten the thick juicy rind.

"That stuff is doubtless indigestible, and has dammed up the loop of the bowel," was his diagnosis of the cause.

In a short time the saline sent him to the stool, and to please me he used the slop jar.

The stool was watery and abundant, and floating on its surface was the peel of that orange, apparently unchanged, gleaming like gold on the dirty, sudsy discharge.

The mass of the protruding hernia disappeared immediately, when the patient returned to his reclining position on the couch.

I advised the gentleman to protect himself by an operation, or by a truss, but he laughed at the idea, and went his way.

---

This month we take pleasure in calling the attention of the JOURNAL's readers to the advertisements of the Foregger Co., of New York; Kora-Konia, of Newark, N. J.; the Merry Optical Co., of Kansas City; Davis & Geck, of Brooklyn, N. Y. These firms are worthy of your patronage and you will do well to investigate their products, if you are endeavoring to keep pace with the progress that is being made in the medical world.

